# Phytochemicals in Cardiovascular and Respiratory Diseases: Evidence in Oxidative Stress and Inflammation

**DOI:** 10.1155/2018/1603872

**Published:** 2018-08-09

**Authors:** Raluca M. Pop, Ada Popolo, Adrian P. Trifa, Luminita A. Stanciu

**Affiliations:** ^1^Department of Pharmacology, Toxicology and Clinical Pharmacology, “Iuliu Hatieganu” University of Medicine and Pharmacy, Cluj-Napoca, Romania; ^2^Department of Pharmacy, University of Salerno, Fisciano, Salerno, Italy; ^3^Department of Genetics, “Iuliu Hatieganu” University of Medicine and Pharmacy, Cluj-Napoca, Romania; ^4^National Heart and Lung Institute, Imperial College London, London, UK

Cardiovascular and respiratory diseases are amongst the leading cause of mortality worldwide. Within these pathologies, excessive oxidative stress and chronic inflammation are important factors with a role in their pathogenesis. Recent studies suggested that natural compounds can be successfully used in order to prevent, control, or block disease progression by targeting oxidative stress and inflammatory mediators. Therefore, this special issue focused on following aims: (i) to understand the mechanisms of initiation and progression of CVD and respiratory disease within the context of oxidative stress and inflammation and (ii) to highlight phytochemical potential in everyday life, by bringing evidence of their properties related to the oxidation and inflammatory processes from CVD and respiratory diseases.

The collection of the review and original research articles in this special issue is approaching a wide range of topics from supplements used in cardiovascular disease prevention or treatment and regulators of cardiovascular diseases to lifestyle interventions like surgical interventions that can potentially reduce cardiovascular risks. Also, new perspectives regarding allergic rhinitis disease management conditions were discussed.

In the original research article, A.-D. Andreicut et al. investigated the phytochemical composition of the flowers, green fruits, and ripe fruits of *Mahonia aquifolium (M. aquifolium)* ethanolic extracts and their antioxidant and anti-inflammatory properties. The *in vivo* anti-inflammatory and antioxidant effects were investigated using a rat turpentine oil-induced acute inflammation model. The authors proved the antioxidant activity and anti-inflammatory effects of the ethanolic extracts by comparing them to diclofenac. All extracts decreased serum nitric oxide (NOx), total oxidative status (TOS), and 3-nitrotyrosine (3NT) and increased total thiols (SH). The variation of malondialdehyde (MDA) was not influenced by the extracts while TNF-*α* was also reduced. Total antioxidant reactivity (TAR) was increased only when flower and green fruit ethanolic extracts were used. Finally, the authors support the use of *M. aquifolium* in the prevention of the inflammatory processes.

Ș. S. Balea et al. investigated the effect of phenolic compounds from fresh and fermented pomace extracts, their antioxidant, and cardioprotective properties using the isoprenaline-induced myocardial ischemia model in rats. Taking into account the actual trend of reducing oxidative stress with herbal supplements or functional foods with antioxidant properties, the authors evaluated the grape pomace in vitro antioxidant activity using the most frequently used method like DPPH test. Following, they further investigated the grape pomace extracts *in vivo* cardioprotective properties by ECG monitoring and measuring creatine kinase, aspartate transaminase, and alanine transaminase serum levels. The *in vivo* antioxidant effects were also evaluated measuring TOS, TAR, MDA, SH, GSH, and NOx serum values. They showed that the high total phenolic content in both fresh or fermented grape pomace extracts presented good *in vitro* antioxidant activity, which improved *in vivo* cardiac and oxidative stress parameters. Overall, their findings suggest that grape pomace extract pretreatment may be used as an option for heart preconditioning.

Next, M. Bertocchi et al. investigated the cytotoxicity, anti-inflammatory, and angiogenic activities of *Boswellia serrata (B. serrata)* extracts on primary culture of porcine aortic endothelial cells (pAECs). Since *B. serrata* is being used in Ayurvedic medicine to treat different diseases with an inflammatory component, the authors of this study aimed to characterize the active molecules of *B. serrata* extracts and then further to evaluate their possible biological effects. Taking into account the great variability of phytochemical concentration and composition encountered in the commercial products, the authors underlined the importance of this aim which should be a prerequisite for testing any biological effect. Further, the anti-inflammatory and angiogenic properties of *B. serrata* extracts were tested using lipopolysaccharide- (LPS-) induced cytotoxicity by comparison with pure 11-keto-*β*-boswellic acid (KBA), 3-O-acetyl-11-keto-*β*-boswellic acid (AKBA), and *β*-boswellic acid (*β*BA), either individually or in different mixtures. They demonstrated that *B. serrata* extracts had a protective effect against LPS inflammatory stimulus in the endothelial cells. In particular, the hydroenzymatic extract presented the best results with cell viability completely restored at all studied doses and was free of cytotoxicity. The comparison of Boswellia extracts with the pure BAs suggested that the anti-inflammatory effect of the extracts was also related to the other bioactive molecules like triterpenes from their composition. Importantly, the authors underlined that proliferative stimulation can also occur instead of the protective effect depending on the used formulations. Thus, when used in humans and animals for its cardiovascular health, the concentration and the composition of the bioactive components should be carefully considered.

In their study, P.-L. Hsu et al. investigated the effect of *Ganoderma lucidum* (GL) in atherosclerosis using a carotid-artery-ligation mouse model. Through their article, the authors aimed to bring evidence regarding GL effect in atherosclerosis, still considered as a leading cause of death disease associated with chronic oxidative stress and inflammation. Being widely accepted and used as herbal supplements, especially in the traditional Chinese medicine, GL is commercially cultivated under controlled conditions with known chemical composition. The authors showed that GL protected the carotid artery from disturbed flow-induced atherogenesis, prevented carotid artery ligation-induced neointima formation, alleviated disturbed flow-induced oxidative stress and proatherogenic response in endothelial cells, suppressed oscillating flow-induced inflammatory response in endothelial cells, and protected endothelial cells against oxidative insults while deferred GT treatment effectively inhibited atherogenesis. Thus, their findings provide evidence that Ganoderma, by its triterpenoid composition, possesses atheroprotective effects and could be used to enhance the Ganoderma supplement health benefits.

The cardiovascular disease problematic issue was also addressed by W. Sun et al. which investigated the potential role of sirtuin 3 (SIRT3) as a new regulator of cardiovascular diseases. They analyzed recent studies referring to the role of SIRT3 in CVD physiology and pathology, with emphasis on its regulating mechanisms in the cardiac processes. The authors aimed to find its prospects in clinical application. The role of SIRT3 in ischemic heart disease, in hypertrophy and heart failure, in the drug-induced cardiotoxicity, in diabetic cardiomyopathy and cardiac lipotoxicity, and in hypertension was discussed. Its protective effect against CVDs was suggested, emphasizing its important role in cellular energy metabolism, in the oxidative stress processes, and in apoptosis. Also, the components extracted from traditional Chinese medicine which promoted SIRT3-mediated cardioprotective effects were described.

A. F. Cătoi et al. investigated the variations of serum chemerin in patients with laparoscopic sleeve gastrectomy (SG) in order to assess its correlation with the inflammatory and nitrooxidative stress markers. They hypothesized and verified if the increased chemerin levels are changing 6 months after SG and whether these changes are influencing some specific inflammatory markers like high-sensitivity C-reactive protein (hsCRP), tumour necrosis factor alpha (TNF-*α*), and nitrooxidative stress (NOx, TOS, TAR, and OSI). They were expecting that these variations could lead to a reduction of the cardiovascular risk. In the end, their study did not confirm significant changes in chemerin levels, neither in other inflammatory and nitrooxidative stress markers, with the exception of hsCRP and OSI levels, further investigations being needed in order to confirm their hypothesis.

Finally, in their study, the allergic rhinitis was approached by I. A. Muntean et al. which is considered an important risk factor for asthma's occurrence. The authors' aims were to target the minimal persistent systemic inflammation in allergic rhinitis investigating the role of H1 antihistamines. Thus the adhesion molecules' profile (intercellular cell adhesion molecule 1 (ICAM-1), vascular cell adhesion molecule 1 (VCAM-1), and E-selectin) was analyzed in patients with allergic rhinitis with focus on H1 antihistamine (desloratadine or levocetirizine) influence on these markers. H1 antihistamine treatment significantly improved the total symptom score and significantly decreased the levels of plasmatic eosinophils and total IgE and the levels of fractionate nitric oxide in exhaled air (FeNO) after 1 month of continuous therapy. Also, the plasmatic levels of ICAM-1 and E-selectin were significantly decreased. This study emphasized the 2nd generation H1 antihistamine anti-inflammatory role, which was demonstrated through CAM plasmatic level reduction, in patients with persistent allergic rhinitis, giving in this way new insights in allergic disease management.

Overall, the papers reported in this special issue highlight the importance of phytochemicals and their role in CVD prevention. Moreover, the attention is focused on our understanding regarding their use in traditional medicine, on their safety and efficacy which are in strong correlation with plant extract composition and their phytochemical concentrations. New inside on better allergic rhinitis disease management is also provided.

*Raluca M. Pop* *Ada Popolo* *Adrian P. Trifa* *Luminita A. Stanciu*

